# How does mentoring occupational therapists improve intervention fidelity in a randomised controlled trial? A realist evaluation

**DOI:** 10.1186/s12874-024-02269-4

**Published:** 2024-07-01

**Authors:** Blanca De Dios Pérez, Jose Antonio Merchán-Baeza, Katie Powers, Kristelle Craven, Jain Holmes, Julie Phillips, Ruth Tyerman, Kate Radford

**Affiliations:** 1 Centre for Rehabilitation and Ageing Research, School of Medicine, University of Nottingham, Nottingham, UK; 2 Research group on Methodology, Methods, Models and Outcomes of Health and Social Sciences (M3O), Faculty of Health Sciences and Welfare. Centre for Health and Social Care Research (CESS), University of Vic-Central University of Catalonia (UVic-UCC), Vic, Spain; 3 Institute for Research and Innovation in Life Sciences and Health in Central Catalonia (IRIS- CC), Vic, Spain; 4 Mental Health and Clinical Neurosciences, School of Medicine, University of Nottingham, Nottingham, UK

**Keywords:** Realist evaluation, Mentoring, Occupational therapy, Vocational rehabilitation

## Abstract

**Background:**

Integrating complex interventions within healthcare settings can be challenging. Mentoring can be embedded within a randomised controlled trial (RCT) to upskill and support those delivering the intervention. This study aimed to understand, from a realist perspective, how mentoring worked to support implementation fidelity for occupational therapists (OTs) delivering a vocational rehabilitation (VR) intervention within the context of an RCT.

**Methods:**

A realist evaluation using secondary data (emails, mentoring record forms, interviews) collected as part of an RCT. Three researchers coded the data following content analysis, focused on refining or refuting an initial programme theory by exploring the interactions between context, mechanisms, and outcomes. The research team met to further refine the programme theories.

**Results:**

Data from 584 emails, 184 mentoring record forms, and 25 interviews were analysed following a realist approach. We developed a programme theory consisting of two contexts (trial set-up, ongoing mentoring), nine mechanisms (collective understanding, monitoring, timely support, positive reinforcement, reflective practice, support data completeness, facilitation strategy, shared learning experience, management of research and clinical duties), and three outcomes (improved confidence, improved fidelity, reduced contamination).

**Conclusions:**

Offering mentoring support to OTs delivering a VR intervention as part of an RCT improves intervention fidelity and reduces the risk of contamination. It improves OTs’ understanding of the differences between their clinical and research roles and increases their confidence and competence in trial paperwork completion and identification of potential contamination issues.

**Supplementary Information:**

The online version contains supplementary material available at 10.1186/s12874-024-02269-4.

## Introduction

Complex interventions are characterised by having multiple intervention components, involve behaviour change from several stakeholders and can influence multiple outcomes [1]. These are commonly used in health settings to influence important health outcomes [2]. One such intervention is vocational rehabilitation (VR), which focuses on supporting people with illness or disability to remain, return to, or find new employment [1, 3].

Implementing complex interventions within healthcare systems is challenging and time-consuming [4]. Several barriers have been identified to deliver complex interventions with fidelity at organisational and professional levels [5]. In a previous systematic review [6], barriers to maintaining fidelity in the delivery of rehabilitation to people with long-term neurological conditions included a lack of training availability and therapists’ lack of confidence in offering new interventions, especially if there is a delay between training and the start of intervention delivery [5, 6]. Implementing new interventions in clinical practice or a clinical trial context may require changes in staff knowledge, skills, confidence, and attitudes, and how they apply new learning in practice is a complex process [5].

To overcome some of these barriers, healthcare professionals delivering complex interventions rely on implementation strategies [7]. These can be defined as methods to enhance an intervention’s adoption, implementation, and sustainability [8]. For example, methods for training and supporting those delivering the intervention, such as mentoring, intervention-specific toolkits, checklists, algorithms, and formal practice protocols and guidelines [8].

Mentoring is an iterative process of intentional relationships between individuals dedicated to the professional and personal growth of one another within a structured program bound by a timeframe and defined objectives [9, 10]. Previous studies have examined and demonstrated the effectiveness of mentoring in implementing complex interventions [9, 10] and how mentoring supports intervention fidelity in the context of a trial [11]. However, the mechanisms underlying this learning process remain unclear.

Realist evaluations provide the methods to explore a programme’s context, mechanisms, and outcomes, which allow for determining how an intervention or programme works, for whom, and under what circumstances [12, 13]. This approach enables an understanding of how the context influences behaviour towards an intervention, which, in turn, impacts the outcomes (i.e., participants’ responses) [12]. Evaluating a complex intervention can be challenging, and realist evaluations can be valuable for understanding implementation and which aspects of an intervention (in this case, mentoring) are effective or not [14]. Realist methods have been previously used to understand how complex interventions such as improving primary healthcare services [15], mentoring [16], and mental health rehabilitation services [17] work. Although more time-consuming than other methodologies (e.g., process evaluation), we selected a realist evaluation to identify the active ingredients of a complex intervention (i.e., mentoring) and gain insight into the factors (mechanisms, context) that underlie the effectiveness of mentoring [14].

This study uses realist evaluation methodology to explore the impact of embedded mentoring for clinical Occupational Therapists (OTs) working in the National Health Services (NHS) of the United Kingdom (UK) delivering an early stroke specialist vocational rehabilitation intervention (ESSVR) for stroke survivors as part of a randomised controlled trial (RCT) [18]. We are interested in understanding:

1. What are the underlying mechanisms by which Occupational Therapist-led mentoring supported the delivery of ESSVR for OTs in the RETAKE trial?
2. What are the conditions, in terms of context and mentoring structure, for whom mentoring works, how, and under what circumstances?
3. What other outcomes are influenced by mentoring for the OTs within the trial context?

## Methods

We conducted a realist evaluation drawing on the reporting standards for realist evaluations (RAMESES II) [19].

### Context

The RETAKE [Return to Work after Stroke] trial involved the delivery of an early stroke specialist VR intervention (ESSVR) commencing within 12 weeks of stroke intended to support participants’ return to work and job retention at 12 months post-randomisation. ESSVR was individually tailored to the stroke survivor’s clinical, personal, and professional needs. ESSVR included assessment of the impact of a stroke at work, educating patients, employers and families about the impact of a stroke at work, identifying reasonable adjustments (e.g., written instructions, specialist equipment) to lessen the impact of stroke, work preparation and maintenance, and engagement of stakeholders (e.g., employer, healthcare professionals), amongst other support [18].

OTs delivered the intervention in the participants’ homes or workplaces, according to preference and need. Two OTs were recruited to deliver the RETAKE intervention in each of 16 trial sites [18]. The OTs recruited to deliver the ESSVR intervention adopted the role of mentees and received mentoring from senior OTs who were not involved in intervention delivery. The mentors were also OTs with stroke-specific knowledge and experience in VR and research processes (e.g., two had completed a PhD).

Mentees were based in different healthcare settings, with varying levels of VR and stroke expertise. OTs delivering the intervention completed an initial 2-day training session, followed by a third day six months later, to deliver the VR intervention and engaged in group monthly mentoring sessions delivered remotely [18].

### Development of initial programme theory

Following the realist evaluation framework, we developed an initial programme theory using a context-mechanism-outcome (CMO) configuration through discussion with the research team [13]. While developing the initial programme theory, we conducted a face-to-face group meeting with the eight research team members (i.e., co-authors) to understand how mentoring was delivered during the trial and their thoughts on the impact of mentoring on the trial. A team member (BDP) led a discussion to understand what it was about the way mentoring was delivered, that made a difference to how the OTs delivered the intervention. The research team was encouraged to provide an explanation of how mentoring worked to test the initial theories the team provided, and refine them iteratively through discussion [20, 21].

The team members had first-hand experience in the trial processes and in supporting the mentees with the implementation of ESSVR. The initial programme theory was:

If research OTs with limited research experience are provided with frequent mentoring during the trial (Context), then they will become more confident in delivering the intervention (Outcome) and deliver the intervention with improved fidelity (Outcome), because mentoring will help them develop an understanding of the need to adhere to the intervention process and trial (e.g., completion of forms) procedures (Mechanism).

We developed a series of secondary programme theories to explore the mechanisms by which mentoring brings about its change in more detail, which allowed us to create a code book for analysing the data. These are presented in additional file 1.

### Data Collection

This study involved secondary data analysis from the mentoring records collected during the trial. The methods used to embed the mentoring in the trial are published elsewhere [22]. The data was collected between March 2018 and April 2020 as part of the trial and included mentoring record forms (*n* = 184) (including content regarding implementation and clinical issues, challenges experienced by the OTs, and issues with the employer’s intervention), emails (*n* = 584) between the trial team and OTs, and interviews with mentors (*n* = 6) and mentees (*n* = 19) at the end of the trial to explore their views and experiences on the trial; these were not conducted following a realist perspective. We obtained informed consent from all participants.

The demographic characteristics of the mentors and mentees involved in the RETAKE trial are reported elsewhere [22].

### Data analysis and synthesis

Interviews were audio-recorded, transcribed verbatim by an external company, and analysed following content analysis. All data (emails, interview transcripts, and mentoring record forms) were systematically coded following a realist evaluation framework. We developed a definition for each of the mechanisms identified in the initial programme theories to allow for consistency during the data coding process, and these were reviewed by the full research team.

Data from each data source (i.e., emails, mentoring record forms, interviews) were coded according to CMO configurations using Microsoft Word, and then data was merged in Excel for synthesis. The context refers to the background where the mentoring was implemented, including the trials and research site environment; the mechanisms refer to how the mentoring worked and what it triggered in the mentees (e.g., changes in feelings or thoughts), and the outcomes were the expected or unexpected consequences of the mechanisms acting in the given context. Only data related to the mentoring processes were extracted, using a bespoke data extraction form (additional file 2).

Three researchers (KP, JM, BDP) were involved in the data coding, and one author (BDP) oversaw the data coding and reviewed the data extraction forms. Discrepancies were discussed between the three researchers, and a fourth researcher (KR) was contacted to resolve discrepancies through discussion.

The first step in data analysis involved three researchers (KP, JM, BDP) reading and coding 10 emails independently to identify mechanisms involved in the mentoring process. The researchers met to discuss any discrepancies and distributed the data to code independently, arranging monthly meetings to discuss any issues with data extraction, new mechanisms identified, refine their definitions, and revise the programme theories.

Data were synthesised following the steps described by Wong et al. [23]. The CMO configurations coded were grouped based on the mechanisms that best explain the relationship between the context and outcomes of the mentoring support integrated within the trial. We developed “if-then-because” statements (i.e., “if” context, “then” outcome, “because” mechanism) to summarise the CMO configurations [23]. The data synthesis followed an iterative process to develop, support, refine, or refute the original programme theories based on the evidence from the mentoring records.

### Team consultation

Following the data synthesis, the research team met to discuss the programme theories and refine them based on their knowledge and experiences of the mentoring and trial processes. Three authors (BDP, KP, JM) presented the programme theories to the rest of the research team who were involved in the trial to ascertain their views on the impact of mentoring on intervention delivery. The research team was presented with all the components of the programme theory and asked to reflect on whether the theories developed were valid or should be refined. Three authors (BDP, KP, JM) took notes on the views of the research team to refine the programme theories.

### Development of a theoretical framework

The data from the mentoring records and consultation with the research team was synthesised to develop a programme theory using CMO configurations to explain how mentoring should be integrated within the context of future trials, which can be accommodated for different settings.

### Ethical approval

The RETAKE Trial received full Ethical approval through the East Midlands–Nottingham 2 Research Ethics Committee (Ref: 18/EM/0019) and the NHS Health Research Authority. This study involves secondary analysis of anonymised data collected as part of RETAKE, and refinement of the programme theory through discussion with the research team involved in trial delivery and mentoring.

## Results

Data from 584 emails, 184 mentoring record forms, and 25 interviews were analysed. Data analysis generated two contexts, nine mechanisms, and three outcomes relevant to mentoring (Table 1). We present first the context relevant to mentoring, followed by the mechanisms and the resulting outcomes.

**Table 1:** Definition of programme theory, with illustrative data

Domain	Theme	Definition	Illustrative Quote
**Context**			
	Trial set-up	The support received before the trial starts allows mentees to develop the necessary VR skills and identify potential barriers arising during trial set-up.	“Both [Trial Manager] and mentor 3 reported that the funding in [research site] had been pulled due to changes in the excess treatment costs process. I reported that [Trial Manager] had said the situation is being escalated internally to try and identify a means to fund the study. Unfortunately, there is nothing we can do at the moment.” [Emails_December_2018; Mentor 1]
	Ongoing mentoring	The interactions between mentors, mentees, members of the research team, and trial sites that occur throughout the study duration.	“I think it’s just – we’ve got the manual, but the reality is, there’s not one participant that’s going to prescriptively follow it, so it’s all the nuance in how to work with specific workplace context. So, it may be to do with – well, even things like the legal aspect of things, about reasonable adjustments or finding – just getting feedback on what you’re doing and knowing you’re doing the right thing.” [Interview OT 5]
**Mechanism**			
	Collective understanding	Developing a collective understanding of the mentees as RETAKE intervention OTs, ensuring they understand their responsibilities, study procedures, and differentiate their usual NHS role from the RETAKE role.	“During mentoring, I did talk about triaging participants if you feel overloaded as opposed to decreasing or stopping recruitment. Recruitment is of vital importance to the study. We want you to telephone the participant within 48 hours as this is the EARLY bit [of the intervention]” [Emails_June2019- Mentor 1]
	Monitoring	Providing longer-term support and responding to changing needs. Offering feedback on mentees’ adherence to research processes and intervention delivery.	“[In mentoring] I said that other OTs were sending me letters to check and they [mentees] were welcome to do that. [Mentee] said she had started writing letters to her participants since the training and feels it …helps everyone “to be on the same page”. [Email_Dec2018; Mentor 3]
	Timely support	Providing support when or before a problem appears (e.g., identifying alternative OTs if a site cannot cope with demand).	“I just used the mentoring then to kind of go over everything to think well okay, I am doing this correctly or what do I need to do? … There are a couple of times where I have had to get support really, really quickly and that has never been a problem as well, so emergency help as well.” [Interview OT 1]
	Positive reinforcement	Mentees are positively encouraged when they are delivering the intervention as intended and demonstrating command of VR (e.g., good clinical decision making). They are also supported when they experience challenges with trial procedures and reminded that it is okay to have difficulties.	“I think most of us probably don’t have… are not working with patients returning back to work all the time so I think sometimes you are not always 100% sure of yourself and what you are doing.” [Interview, OT 36] ‘And she [mentor] would check letters and things before we sent them out to make sure we were on the right lines with things and give us some tips for writing them initially.’ [Interview, OT 8]
	Reflective practice	Mentor or mentees’ ability to think about evaluate and implement trial processes and procedures, and act based on their previous experiences.	‘But she [OT mentor] did it in a way where she would sort of help us to answer the question rather than just telling us the answer, so she would make us think about it.’ [Interview OT 8]
	Support data completeness	Supporting completion of case report forms, study documentation and reviewing letter-writing standards for documenting trial intervention delivery.	“Hi [mentor 3], Sorry I have another question. On form 22 which is the session content form, I am wondering what to do with the graded return to work with my participant. As he is self-employed, do I tick RTW without direct employer contact?” [Email_September_2018 Mentee 10]
	Facilitation strategy	Mentors find and connect mentees with key stakeholders based on the identified problem.	“I will ask [mentor 1] to liaise with and advise the OTs. So long as they manage the list and prioritise within it, this shouldn’t be a problem. Hopefully, the intervention allocation will change, and the waiting list will then be reduced. There could then be a period where hardly anyone is allocated to the intervention and pressures will ease. Please let me know who I can talk to regarding the service development. If it does go ahead my big ask would be not to deliver VR to people randomised to usual care who have consented to participate in the trial.” [Emails_June_2019; Research Team – CI]
	Shared learning experience	Mentors share their experience and best practice. Mentees generate a peer support group that improves their self-efficacy.	“Mentor 1 kindly presented a case study. A man who was in hospital Sat-Thurs, she contacted him the day he left hospital and saw him a week later. He returned to work on the Monday after discharge as money was an issue. She persuaded [him] to take another week of sick, wrote a sick note and met with his employer. She has planned a GRTW and mentees 3 and 4 asked if mentor could share the letter to employer as they are keen to learn.” [Emails_Sept_2018; Mentor 1]
	Managing research and clinical duties	Mentees learn to manage the trial intervention delivery and administrative burden of the project alongside their usual clinical tasks and responsibilities.	“Just to confirm that you told me you are doing all of the screening and recruitment of participants as well as holding a caseload for RETAKE. Plus you still have all your usual NHS responsibilities, which are also huge. L, the research nurse is doing some screening but not to the detail you are and she also as 6 other trials, so it sounds like she cannot do anymore to assist you. If you need us to approach L and support her in any way, we can.” [Emails_Sept_2028; Mentor – 5]
**Outcome**			
	Increased mentee confidence	Mentees developed research and clinical (i.e., vocational rehabilitation) skills that allowed them to trust their ability to fulfil their role as a RETAKE OT.	“I have seen my first patient for an initial assessment. Just wanted to check I’m doing the right things, and ask a few questions.” [Emails_January_2020; Mentee 3]
	Intervention delivered with fidelity	The intervention was delivered as intended. Mentees delivering the intervention accurately and improved the recording of the support provided.	“I think they – firstly, I think they’re there to monitor and check that we’re actually delivering the intervention. I think it’s to ensure that we’re – for the research, we’re doing what we should be doing.” [Interview, OT 5]
	Reduced contamination	Mentees and the research team were able to identify instances when changes in services, staffing and new research studies might lead to changes in the outcomes of interest in the trial, therefore identifying risk of contamination.	“Dear all, please see this email below…It seems there is another study wanting RTW participants in [city]. I do not think we have any sites in West Midlands. But it is yet another potential factor affecting results in [city] so needs recording as a potential contamination” [Emails_July_2019; Email between mentors and CI]

OT: Occupational therapist; RTW: Return to work; CI: Chief Investigator; MRF: Mentoring record form

### Context

We identified two main themes within the context domains: trial set-up and ongoing mentoring sessions.

#### Trial set-up

Mentors started engaging with the mentees when a site was recruited to the trial. Some mentors were involved in training the mentees in ESSVR. These interactions were essential for the mentors to understand the mentees’ previous experiences (i.e., who had prior VR experience and who did not), clinical responsibilities at each site and the mentees’ critical areas for development.

#### Ongoing mentoring

During the trial, mentoring involved monthly group sessions and guidance to follow trial and ESSVR procedures. Mentors also provided ad hoc mentoring sessions for those who needed additional support. Mentees reported challenges around managing clinical and research requirements, such as insufficient time at work to deliver the intervention to trial patients, NHS managers expecting OTs to support the same number of patients as before the trial, and support needs in completing trial documentation and letters to other stakeholders (e.g., employers).

### Mechanisms

We elicited eight main mechanisms to describe how mentoring worked in the context of the RETAKE trial.

#### Collective understanding

One of the main mechanisms identified was “collective understanding”. Mentors supported mentees to understand the essential ESSVR components and tasks related to the documentation of the trial. Over time, mentors and mentees developed a relationship of trust that made mentees more likely to ask for support. This relationship matured as mentors shared examples from their experiences and were understanding and open-minded with the mentees’ questions.

As mentees developed a collective understanding of their research and clinical role, they became more accurate in identifying instances of potential contamination and delivered ESSVR with improved fidelity. Problems developing this collective understanding led to delays in study set-up, instances of contamination, and mentees lacking awareness of ESSVR procedures (e.g., how to discharge a participant). This mechanism was a driver for all other mechanisms within the trial.

#### Monitoring

This mechanism relates to the mentors’ ability to review progress made and provide long-term support to mentees during the trial.

At the trial set up stage, if the mentors engaged with the mentees to track progress on the sites, then this facilitated the identification of any changes in the trial sites that might lead to contamination (e.g., new service developments or services, identifying new or competing research studies, etc.). Mentors were then able to prepare the mentees to engage with the trial (e.g., access to trial systems and paperwork, data protection and Good Clinical Practice (GCP) training), and the mentors were able to identify those in need of further training. For this to occur, the mentors had to be proactive in developing trusting relationships with the mentees, potentially through the initial training that the mentees received. This was reinforced over the course of the trial due to further interactions during the mentoring sessions. This enabled the mentors to monitor progress, identify potential problems, and address the mentees’ concerns.

During the intervention delivery period, this mechanism related to mentors reassuring mentees that they were on the right track with the intervention delivery, which increased the mentees’ confidence in their ability to deliver the support, especially those who were new to VR. Mentors also shared summaries of the content discussed in the group mentoring sessions and arranged further 1-to-1 sessions for those who needed additional support. This allowed the mentees to have a re-accessible resource to review in their own time.

#### Timely support

In each group mentoring session, mentees were invited and actively encouraged to share details about their current caseloads. Mentees were especially encouraged to share instances and situations where they were struggling. Through sharing these struggles with the mentoring group, the mentees could seek advice from their mentors and peers and learn from their mistakes before integrating them into ESSVR delivery. Mentors were also able to identify their mentees’ further training needs in a timely way. This helped solidify skill development, increase confidence in delivering ESSVR, and ultimately helped facilitate fidelity to ESSVR.

Mentors reflected that even when mentees had developed greater confidence, the mentees still sought timely support, but were often looking for reassurance rather than solutions to a problem. The mentors also reflected that the more mentees engaged in disclosing their struggles in mentoring and subsequently received support, the more likely they were to disclose their struggles in future sessions.

It is important to consider that early identification of issues relied almost entirely on the mentee communicating their experience of delivering ESSVR. Throughout the trial process, situations arose where mentees required timely input from their mentors, often between mentoring sessions. When mentees were not able to disclose their struggles within their monthly mentoring session, mentors encouraged the mentees to contact them for individual support. In some circumstances, mentees did not engage in some of the monthly mentoring because of environmental factors, such as busy caseloads and personal circumstances. Mentors highlighted that, at times, providing timely support to mentees seeking individual support was difficult as the timeliness was dependent on the workload of the mentor and the mentee.

#### Positive reinforcement

When mentors provided reassurance about a mentee’s proposed course of action, mentees felt valued (and therefore disclosed more). Still, they were also reassured in their actions, which helped solidify their skill development, ESSVR knowledge, and confidence. When mentees are more confident in ESSVR delivery, they were more likely to go outside of their comfort zones to engage and liaise with employers.

Increases in mentees’ ESSVR delivery and research self-efficacy also increased the mentors’ confidence in the mentees’ abilities. Increased confidence in the mentees meant that the mentors could plan their time to give more support to less confident mentees. Mentors reflected that increased confidence among the mentees led to the group mentoring becoming more peer-led, which, in turn, led to the mentees feeling more valued.

Some mentees became more dependent on their mentors due to positive reinforcement. Mentors provided a safety net for mentees with little to no experience in VR or research. There was also a shared sentiment that, in some cases, mentees stopped coming to group mentoring, because they preferred the positive reinforcement and increased self-efficacy from one-to-one mentoring sessions. For these mentees, mentors reportedly had to delay their response rate to foster independence.

#### Reflective practice

ESSVR was a new intervention for the OTs in the RETAKE trial. Its components pushed the mentees beyond their comfort zones. In group and individual mentoring sessions, mentors provided a space for mentees to think about how they might approach ESSVR and other trial-related problems. Through evaluating the ESSVR process and trial procedures and discussing previous experiences, mentees were able to learn from each other and develop shared ‘best practice’. By sharing each other’s cases and reflecting on those cases, mentees were able to build their knowledge of how to individually-tailor ESSVR to their participants’ needs. This facilitated increased confidence and self-efficacy in both ESSVR delivery and trial procedures.

For many OTs, participating in a monthly, hour-long reflective practice session was an opportunity they did not have in their usual care roles, even though was written into some OTs’ contracts.

#### Support data completeness

This mechanism refers to support in completing forms, recording study data, and training and developing letter templates to communicate with participants and other stakeholders.

During mentoring sessions, mentors emphasised the importance of accurately completing study documentation and intervention records. They offered support with completing forms, which increased the mentees confidence and competence for accurate completion.

More reliable data were obtained from mentees who completed forms and reports immediately following ESSVR delivery. Mentees who completed forms later tended to rely on their clinical notes and were less accurate in their reporting.

#### Facilitation strategy

On numerous occasions, the mentees raised problems with the mentors that they did not know how to solve by themselves. For example, how to engage with managers, support recruitment or understand problems with how they were being paid to deliver the research. Their relationships with the mentor made them more likely to contact the mentors or other research team members to discuss issues and seek advice. For example, the mentors contacted the trials unit or the Chief Investigator to address complex trial issues, the mentors supported mentees on RETAKE administrative duties to relieve staff pressures, or mentors and mentees worked together to understand how to contain contamination at a site. This interaction improved intervention fidelity and allowed the development of a network of stakeholders with different skills to address each problem.

Overall, the mentors acted as case managers finding solutions to problems or identifying stakeholders who could address the problem. The mentees saw themselves as key members of the trial, with a role in ensuring the intervention was delivered as intended to ensure a robust trial outcome. Mentors themselves were able to address recurrent trial challenges such as low recruitment or staffing issues because they had gained this experience through the trial and knew who to contact for each problem.

#### Shared learning experience

This mechanism evolved over the study duration. The RETAKE trial lasted 6.5 years with 47 months of recruitment and an extra 12 months of ESSVR delivery. It was also impacted by the pandemic; thus, there were many changing circumstances that required shared learning to address problems. However, mentoring was seen as a highly acceptable process in the trial.

During the trial set-up, mentees got to know each other at the face-to-face intervention training and site initiation visit sessions. These trial set-up interactions, together with interactions in their own trial sites and during mentoring, allowed them to develop working relationships beyond their role in the trial.

During mentoring, mentors took a leading role and shared their own experiences and best VR practices with the mentees. Over time, mentees became more confident in offering each other support, creating an environment of peer support where they discussed common issues and potential solutions to barriers based on their experience. Mentees also discussed what they had learned and what they found most helpful. For example, mentees discussed how they learned by “doing” (i.e., delivering the intervention), and having a mentor to answer questions helped boost their confidence and knowledge in VR. Mentees were also grateful that other mentees were sharing initiatives and procedures followed at their site, which could be integrated into other sites to improve how services are delivered. This approach helped mentees learn from one another and build relationships over time.

#### Management of research and clinical duties

This was the only identified mechanism that affected delivery of the intervention with fidelity.

The Covid-19 pandemic had a direct impact on the mentees usual care clinical duties, which by extension, limited their time available to deliver ESSVR and engage in the mentoring. This was particularly challenging for mentees when either they or someone in their NHS teams contracted Covid-19, and were unable to work. The increased workload meant mentees could not deliver the intervention.

Mentees also experienced challenges associated with their NHS line managers being unsupportive of their research role. Despite mentees being paid indirectly via Excess Treatment Costs [24] to deliver ESSVR, within site transfer of these finances into therapy services’ budgets, research naivety in some NHS sites (in both line managers and research and innovation), together with local recruitment issues, often meant the OT mentees research time was not backfilled and some mentees were expected to deliver the research activity in addition to their usual clinical role. This resulted in mentees feeling overwhelmed.

If mentees were experiencing problems managing their clinical and research duties, they typically experienced problems with intervention delivery, and following research procedures.

### Outcomes

We identified three main outcomes by integrating the mentoring sessions into the trial.

#### Improved confidence

Mentees became more confident in their ability to engage in research and deliver ESSVR as expected, developed skills to contact other stakeholders (e.g., employers, occupational health representatives, etc.), and engaged in conversations to problem-solve challenges arising during the intervention. This improved confidence resulted from the shared learning experience and open-minded mentor and mentee support. Mentors also gained confidence in the mentees ability, based on the questions they asked and their approach to solving identified problems. Mentees who overestimated their knowledge and ability to deliver ESSVR engaged in fewer mentoring sessions, and therefore, had fewer opportunities to reflect on their practice.

#### Improved fidelity

Through the mentoring sessions and contact with the mentors between sessions, mentees became more familiar with the clinical aspects of ESSVR delivery and research processes (e.g., how to record the intervention content). Therefore, they became more accurate in delivering and reporting ESSVR content. Therefore, they became more adherent to the individual components of ESSVR and more accurate in their reporting of ESSVR component delivery. OT mentors found fewer discrepancies between the intervention delivered and the intervention recorded, and OT mentees became more accurate in differentiating between intervention components and following procedures according to the different trial circumstances.

#### Reduced contamination

The topic of contamination was addressed during the trial set-up training sessions to allow mentees to identify potential issues that may arise during the lifetime of the trial.

As mentees became more familiar with the intervention manual and research processes, they became more aware of changes in their NHS services and local centres that may lead to contamination.

Mentees were responsive in communicating these changes to mentors and the research team, which allowed them to implement solutions to overcome contamination issues.

### Revised Program Theory

**Fig. 1:**
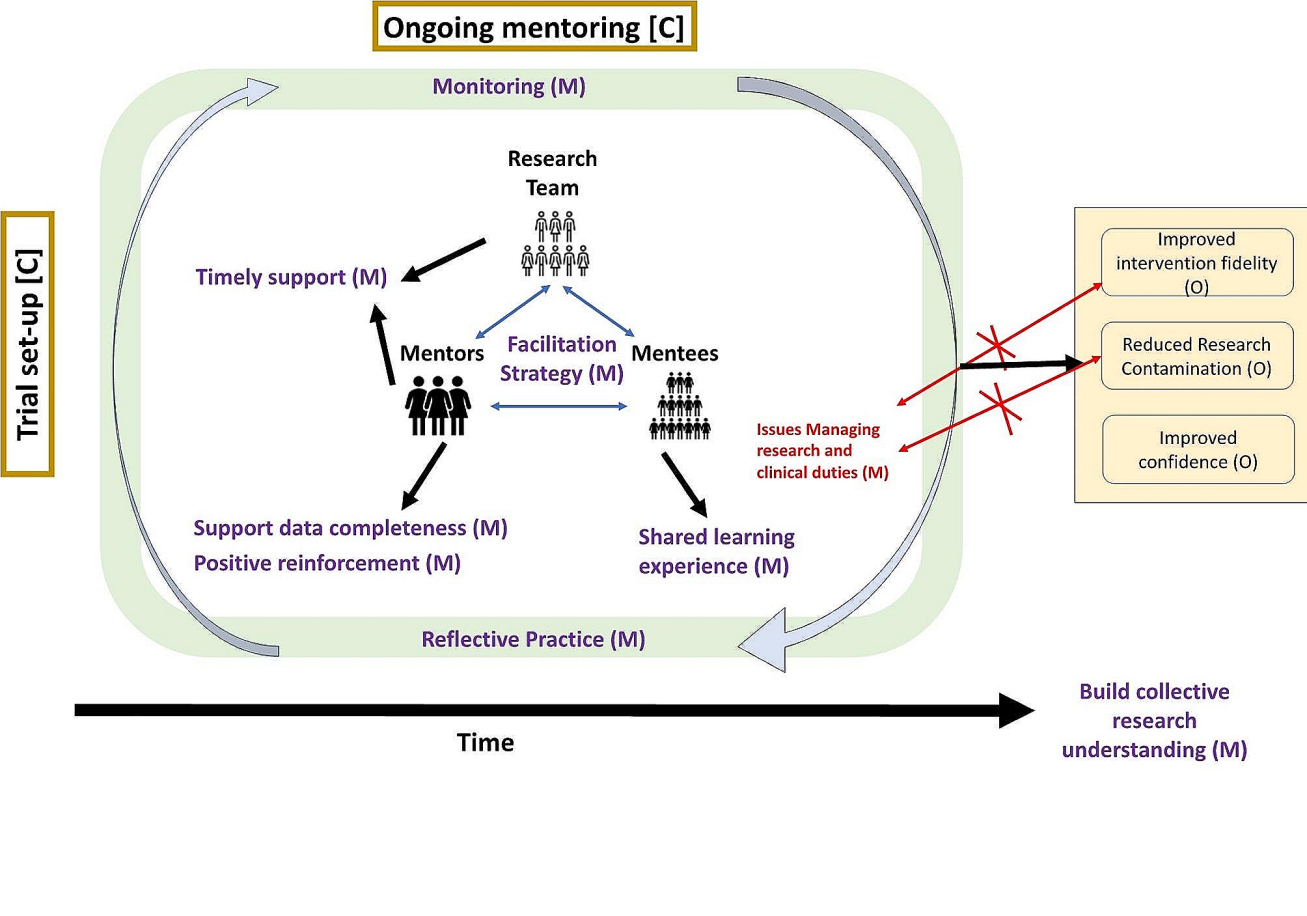
Mentoring programme theory. Legend: Red arrows depict barriers to achieving outcomes

Based on the data analysis, a visual representation of the revised programme theory representing the interaction between the different CMOs is presented in Fig. 1.

The context related to the “trial set-up” stage is at the programme theory outset. It refers to the starting point when the mentees and mentors got to know each other, shaping relationships and by extension, their perceptions of mentoring. Mentors received training on their role and expectations. All had worked as mentors on previous trials and had prior expertise in VR. The second context, “ongoing mentoring”, is located across the logic model to reflect changes over time.

The underpinning mechanisms of mentoring are presented as interacting with each other to reflect the ESSVR intervention and research learning throughout the trial.

The programme theory describes how embedding mentoring within the trial led to the proposed outcomes. One outcome (improved confidence) impacts at an individual level (mentees), and it is essential to achieve the intervention-related outcomes (improved fidelity and reduced contamination).

## Discussion

This study aimed to develop a realist programme theory to explain how mentoring supports implementation fidelity in a complex intervention trial. We identified nine mechanisms (collective understanding, monitoring, timely support, positive reinforcement, reflective practice, support data completeness, facilitation strategy, shared learning experience, management of research and clinical duties) and three outcomes (improved confidence, improved fidelity, reduced contamination) that describe how mentoring helped clinical OTsdeliver a complex VR intervention. The programme theory illustrates a process where mentees become more knowledgeable in ESSVR and trial processes, which helped them deliver the intervention with improved fidelity and identify problems (i.e., contamination).

Our previous research exploring the impact of mentoring in the context of the RETAKE trial showed that mentoring could improve fidelity and that it was valued by both mentors and mentees [5]. However, it was unclear how mentoring works in a trial context. This realist evaluation suggests mentoring helped the mentees gain confidence in their clinical and research skills. This enabled them to acknowledge when they needed further support and helped them identify possible deviations from the trial protocol. The relationship between mentor and mentees was important for the success of mentoring because mentees felt comfortable discussing the challenges they were facing with the mentors without feeling judged about their abilities.

Using realist evaluation has provided valuable insights into how trusting relationships between the mentors and mentees, which developed throughout the trial, exposed where mentees required further support to implement the intervention. Notably, the realist evaluation revealed that mentees faced greater challenges with the research-related aspects and balancing their clinical and research responsibilities than with delivering the VR itself. Additionally, mentors acted as support staff, managing the practicalities of combining research with clinical practice, which was essential for the OTs to deliver the trial intervention as intended. These insights into generative causal mechanisms may not have been exposed using other research methods [25].

Developing a collective understanding of the intervention was essential to the success of the trial. It is common for experienced OTs to make decisions in clinical practice based on their previous experiences and expert knowledge [26]. In the context of a trial, for the intervention to be delivered with fidelity, the OTs had to understand the research processes and deliver the intervention “as intended” following a predefined process. Mentors played a critical role in the development of a collective understanding by reiterating practices, and exploring, from the perspective of mentees what their challenges and fears were. This suggests that integration or collaboration between the research and the clinical team is a factor contributing to implementation fidelity, identifying instances of contamination, and development of a shared vision for trial success.

Mentoring in this trial also offered the OTs an opportunity for reflective practice. While this is an essential part of clinical practice [27] and a requirement of continuing professional development [23], it is poorly practised due to increasing workload pressures in routine care [27]. The RETAKE trial offered protected time for mentoring and reflection. The mentors also adopted a flexible and personalised approach that allowed OTs to seek advice outside the formal group mentoring sessions. This benefitted those OTs with high workloads related to the trial or participants with complex needs that needed further discussion.

The NHS constitution supports the idea of making research accessible to all patients accessing its services [28]. However, this is not always without challenges. Managing research and clinical duties was challenging for the clinical OTs, leading to barriers to delivering the intervention and threatening fidelity. This mechanism was present when the managers were not supportive of the research time because of the use of the clinical OT time on research instead of clinical duties or when the clinical demand was excessive. These are common findings in the literature that led to issues with recruitment and health professionals unaware of how to balance their research and clinical duties [29, 30]. Therefore, it is important for trials to engage all key stakeholders, including NHS managers, in the pre-trial stage to set expectations and manage responsibilities.

Overall, the mechanisms and outcomes identified align with the literature on the topic. In fact, there is evidence that engaging in mentoring leads to intervention fidelity in the context of a trial [11]. There is also evidence that when healthcare professionals receive mentoring, they become more knowledgeable and skilled, and are more likely to follow clinical guidelines [31].

There were, however, barriers associated with attending the mentoring sessions. The most common issues were conflicting work schedules (i.e., clash with clinical duties or patient visits) and technological difficulties (i.e., lack of internet or telephone). Barriers to attending the mentoring sessions reflect the reality of competing clinical and research duties. To overcome these challenges in the trial, mentors offered flexible 1-to-1 support sessions for those who could not attend group sessions or needed additional support. The mentees found this approach beneficial to complement their training needs. This aligns with the literature that supports a combined approach to mentoring (group + individual sessions) to further develop healthcare professionals’ skills [32].

### Strengths and limitations

This study’s main strength is the novel approach to exploring the impact of mentoring from a realist perspective and the extensive data available to understand how mentoring supports intervention delivery in the context of a trial.

Three researchers were involved in the data analysis and synthesis. Although the findings were discussed in detail with the research team, including several mentors involved in the trial, mentees were not contacted to verify the findings from their perspective. Another limitation relates to the generalisation of the findings, which are particular to acute stroke and stroke rehabilitation services.

### Implications for practice and research

Clinical OTs might benefit from having protected time to engage in group sessions to reflect on their practice to learn from their errors and improve the quality of their services within the context of a trial.

Future trials should consider embedding mentoring to aid the early identification of issues and to support therapists in delivering interventions as intended. Future research should also explore what attributes lead to mentees benefitting more from group or individual mentoring sessions to tailor the mentoring to the mentees’ needs. Future research should also examine the long-term impact of mentoring and whether increased skills and confidence leads to OTs becoming more independent in their work t.

## Conclusion

This study provides further evidence on the impact of mentoring in the context of a trial, by developing a theoretical understanding of how and why mentoring works. This realist evaluation offers insight into how clinical OTs can be upskilled in VR and research procedures, improving intervention fidelity, and reducing contamination.

The evidence suggests the need to build a relationship with clinical OTs delivering trial interventions before the trial to facilitate the study set-up and identify further training needs. There is also a need for continued and open communication between mentors and mentees to address questions and build their confidence in their ability to deliver the intervention and engage in the trial.

Embedding mentoring within a clinical trial has the potential to improve intervention fidelity. This is beneficial for improving the interpretation of research outcomes and facilitating the translation of research evidence into practice.

### Electronic supplementary material

Below is the link to the electronic supplementary material.

Supplementary Material 1

Supplementary Material 2

## Data Availability

The datasets used and/or analysed during the current study are available from the corresponding author on reasonable request.

## Abbreviations

CMO: Context-Mechanism-Outcome
ESSVR: Early Stroke Specific Vocational Rehabilitation
NHS: National Healthcare Service
OT: Occupational therapist
RCT: Randomised controlled trial
UK: United Kingdom
VR: Vocational rehabilitation
