# Supplementary material for: How does mentoring occupational therapists improve intervention fidelity in a randomised controlled trial? A realist evaluation
Source: BMC Med Res Methodol. 2024 Jul 1;24:142. doi: 10.1186/s12874-024-02269-4 (PMC11218321; doi:10.1186/s12874-024-02269-4)
Supplement: Supplementary file 1 — Supplementary Material 1 [file 12874_2024_2269_MOESM1_ESM.docx]

**Additional File 1**

Secondary Initial Programme Theories

Legend: C: Context; M: Mechanism; O: Outcome; OT: Occupational Therapist

- If Research OTs work on sites where managers **are more experienced with research, and understand the potential benefits of the trial (C)**, then, they will **attend more mentoring sessions** **(O),** because the managers foster **collaboration and engagement** in the research activities **(M).**
- If Research OTs experience **high levels of workload (C),** then, they will not be able to **attend all mentoring sessions (O),** because they **feel overwhelmed and lack time** for the mentoring **(M).**
- If research trials **provide research OTs with regular mentoring (C)** sessions, then, the mentees will **feel supported (O)** and **more engaged (O)** in the trial, because the mentors can **identify problems before/as they arise [timely support/ early identification] (M)**.
- If mentoring is provided in a **group setting** **(C)**, then, the research OTs will feel more comfortable **sharing their problems (O)**, because the group setting fosters a **relationship of trust (M)** with the mentor and a **sense of belonging (M)** for the research OTs.
- If mentoring is provided in a **group setting (C),** then research OTs will **gain clinical guidance with intervention delivery (O)** and **share their knowledge (O)** to address issues that other research OTs are experiencing, because the group setting **enables a shared learning environment (M) facilitated by the mentor (M).**
- If mentoring sessions **guide the completion of research data collection forms** **(C)**, then the research OTs will be **more accurate in measuring the intervention delivered (e.g., content, dose) (O), return research data within the given timeline (O),** and **improve the overall quality of data collection**, because the mentors **monitor (M)** data collection and support research OTs to **reflect (M)** on how to complete the forms.
- If research OTs **attend mentoring sessions regularly (C)**, then, they will **deliver the** **intervention with fidelity (O),** because they develop a clear understanding of the intervention and trial processes and their role in it **(shared understanding/ believe in RETAKE) (M).**
