# Supplementary material for: How does mentoring occupational therapists improve intervention fidelity in a randomised controlled trial? A realist evaluation
Source: BMC Med Res Methodol. 2024 Jul 1;24:142. doi: 10.1186/s12874-024-02269-4 (PMC11218321; doi:10.1186/s12874-024-02269-4)
Supplement: Supplementary file 2 — Supplementary Material 2 [file 12874_2024_2269_MOESM2_ESM.docx]

**Additional File 2**

Mentoring Data Extraction Form

**Data Extractor Initials:**

**Resource Used:** Email / Interview training experience / OT Transcripts retake experience / Mentoring records Form/ case study interviews / Other:

**Date of Resource:**

**Relevance**

| **Does the document include any data that might be relevant to our programme theories? Which ones?** | |
| --- | --- |
| **Programme Theories** | **Reasoning- Does the document help with improving the programme theory? To what context and outcome are these mechanisms linked?** |
| Early identification/ Timely Support |  |
| Relationship of trust/ Trust |  |
| Positive Reinforcement |  |
| Monitoring |  |
| Reflection |  |
| Shared understanding/ Believe in RETAKE |  |
| Sense of belonging |  |
| Collaboration/ Engagement |  |
| Problem solving |  |
| Other: |  |

**CMOs IDENTIFIED/REPORTED**

Is there any evidence in the data (e.g., interview transcript, email, mentoring record form) that provides data that can be interpreted as a Context, mechanism (resource/response) or outcome? What are the CMOs [Context-Mechanism (resource)-Mechanism (response)-Outcome Configurations (CMOC)] for the data?

| **Context** | **Resource/**  **Intervention** | **Response/**  **Mechanism** | **Outcome** | **Mechanism Level** | **Page reference** |
| --- | --- | --- | --- | --- | --- |
|  |  |  |  |  |  |
|  |  |  |  |  |  |
|  |  |  |  |  |  |
|  |  |  |  |  |  |
| **Mechanisms Level:** Micro = individual (mentor OR mentee OR Therapist); Messo = team (research team, or mentor + mentee as a team, or NHS team); Macro = organisation level (hospital, clinic, workplace, university). **Cells Coloured in Orange:** Context where the mechanism may not fire; Document Reference. | | | | | |

**Other Comment**

| **Other comments about the CMOs** |
| --- |
|  |
